# Simulating photoacoustic waves produced by individual biological particles with spheroidal wave functions

**DOI:** 10.1038/srep14801

**Published:** 2015-10-07

**Authors:** Yong Li, Hui Fang, Changjun Min, Xiaocong Yuan

**Affiliations:** 1 Institute of Modern Optics, Key Laboratory of Optical Information Science and Technology, Ministry of Education of China, College of Electronic Information and Optical Engineering, Nankai University, Tianjin 300071, China; 2 School of Mechanical Engineering, Jinzhong University, Jinzhong 030619, China; 3 Institute of Micro and Nano Optics, Key Laboratory of Optoelectronic Devices and Systems of Ministry of Education and Guangdong Province, College of Optoelectronic Engineering, Shenzhen University, Shenzhen 518060, China

## Abstract

Under the usual approximation of treating a biological particle as a spheroidal droplet, we consider the analysis of its size and shape with the high frequency photoacoustics and develop a numerical method which can simulate its characteristic photoacoustic waves. This numerical method is based on the calculation of spheroidal wave functions, and when comparing to the finite element model (FEM) calculation, can reveal more physical information and can provide results independently at each spatial points. As the demonstration, red blood cells (RBCs) and MCF7 cell nuclei are studied, and their photoacoustic responses including field distribution, spectral amplitude, and pulse forming are calculated. We expect that integrating this numerical method with the high frequency photoacoustic measurement will form a new modality being extra to the light scattering method, for fast assessing the morphology of a biological particle.

Quantitative evaluation of the morphology of biological particles such as cells and organelles provides useful and sometimes critical information for understanding their biological functions and also malfunctions associated with diseases. For examples, the presence of spherical-shaped red blood cells (RBCs) found on the peripheral blood smear indicates an inherited disorder called hereditary spherocytosis^1^, and the enlargement of cell nuclei is often observed for cancer^2^. Ideally, it requires a label-free optical imaging method with high resolution to directly acquire the shape and size of biological particles in their natural state. Photoacoustic imaging represents such a method which exploits the intrinsic light absorption property of biological particles and has been quickly expanding its horizon in imaging at the minimus-scale end: from individual RBCs, epithelia cell nuclei to intracellular melanosomes^3^,^4^,^5^,^6^,^7^,^8^.

The photoacoustic flow-cytometry based techniques, which can detect individual biological particles in sequence, have also been developed recently^9^,^10^,^11^ to address the practical requirement that it is usually a large number of biological particles needed to be examined. In order to rapidly assess the morphology of a biological particle under such circumstance, the better choice is not to image but to model biological particles as the particles with a specified shape to facilitate the size analysis. This strategy has been recognized and applied recently in a recent serial of investigations with high-frequency photoacoustic microscopy (PAM), where the resolution in particle sizing does not come from the imaging resolution (there the approach of acoustical-resolution PAM instead of optical-resolution PAM^3^,^4^,^5^ has been applied) but rather depends on the measure and analysis of power spectra or the angle dependent power spectra of the photoacoustic waves^12^,^13^,^14^,^15^,^16^. The analysis is based either on the spherical model^17^,^18^ or on the FEM (finite element model)^19^.

Although the both models take their own characteristics—the spherical model results in a simple mathematical expression while the FEM can take account of any particle shapes, their respective limitations are also apparent. The experiments have shown that the spherical model for RBCs is only suitable for the photoacoustic frequency up to 100 MHz^13^,^14^,^15^. On the other hand, the calculation based on FEM requires a large computer memory and is usually time consuming, making the simulation on the sequence of biological particles difficult. Moreover, the FEM performs the spatial and temporal discretization process directly on the original photoacoustic wave equation thus is insufficient in providing physical insight about the impact of each parameter.

This situation can be circumvented to some extent by employing the spheroidal model we recently developed^20^. As illustrated in Fig. 1, when considering the photoacoustic wave production of individual RBCs or cell nuclei due to the laser illumination at a typical wavelength locating inside the absorption band of the biological particles (green laser for RBCs^6^ and ultraviolet laser for cell nuclei^8^), we can approximate a normal biconcave-disc shaped RBC as an oblate spheroidal droplet and a cell nucleus as a prolate spheroidal droplet, and then put them respectively into the corresponding spheroidal coordinate systems to solve the photoacoustic Helmholtz equation [Eq. (1) in Method]. Compared to the spherical model, working with a spheroid can take account the angular dependence of the generated photoacoustic wave. Compared to the FEM, the spheroidal model not only yields an analytic solution expressed with spheroidal wave functions (SWFs)^21^ which conveys a wealth of physical information, but also affords a straightfor*w*ard numerical calculation route based on numerically calculating SWFs.

In this paper, we describe such a numerical calculation method and demonstrate various results for characterizing the photoacoustic wave produced by RBCs and MFC7 cell nuclei. To the best of our knowledge, although the numerical calculation of SWFs was used in more difficult problems of the sound scattering and also the light scattering of a spheroidal particle a long time ago^22^,^23^, introducing it into the simulation of the spheroidal particle photoacoustic wave generation has not been reported. The merit of this new application is that only scalar solutions need to be considered and the solutions are not expanded on the mode number related to the azimuthal angle. We envisage that similar to the role played by the T-matrix calculation in solving the light scattering problems of spheroidal biological particles^24^,^25^, the SWFs based numerical calculation will become important for photoacoustic study of biological particles under the spheroid approximation.

## Results

### RBCs suspended in blood plasma

After fully establishing the numerical calculation method through the validation study (see Numerical Verification subsection in Method), we first carried out the detail numerical study on RBCs. We approximate a RBC as an oblate spheroid droplet with the physical properties as listed in Table 1 (explained in Method), and simulate the situation of RBCs suspended in blood plasma. The mass density and the acoustic speed of blood plasma have been set respectively as *ρ*_f_ = 1000 kg/m^3^, *v*_f_ = 1520 m/s by following reference^13^.

Figure 2 shows various results including the photoacoustic wave field distributions (defined here as the spatial distribution of photoacoustic wave amplitude) at three different frequencies [Fig. 2(a–c)], the photoacoustic wave amplitude versus frequency curves for the near field as well as the far field at three typical angular directions [Fig. 2(d,e)], and the photoacoustic pulses formed at the far field [Fig. 2(f)] corresponding to the spectral amplitudes shown in Fig. 2(e). A specified normalization procedure (described in Numerical Calculation subsection in Method) has been exerted for all of these results.

**Figure 1:**
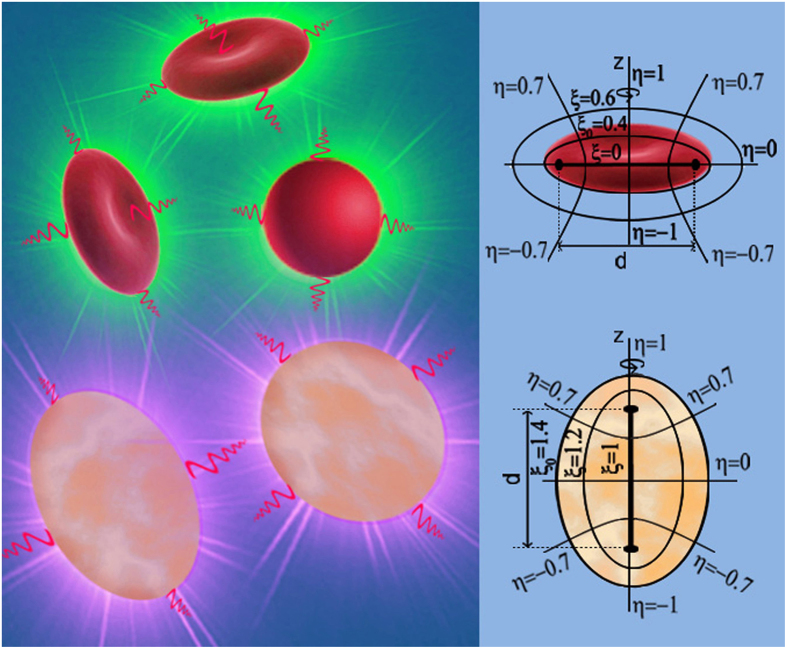
Illustration of photoacoustic wave production of RBCs and MFC7 cell nuclei, the corresponding oblate spheroidal coordinates system for a RBC and prolate spheroidal coordinates system for a cell nucleus. The photoacoustic waves (shown as the red waves) of individual biological particles are produced due to the laser uniform illumination on each RBCs (as the green flashing) and on each cell nuclei (as the ultraviolet flashing) at the respective absorption wavelength (green for RBCs and ultraviolet for cell nuclei).

**Figure 2:**
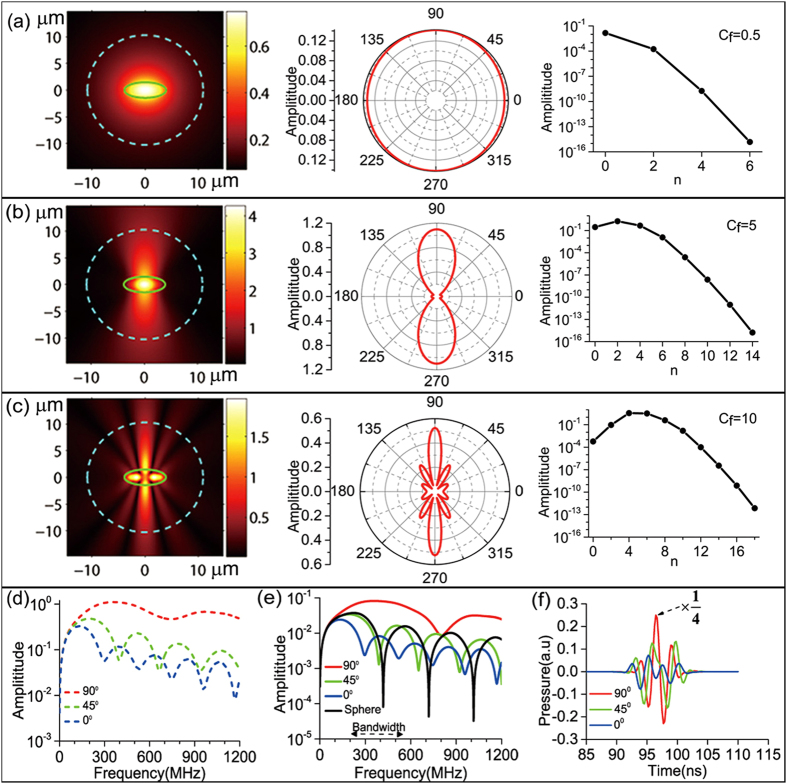
Numerical results of simulating RBCs suspended in blood plasma. (**a**–**c**) Field distribution, polar distribution, and amplitude of the expansion coefficient 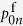 at three different frequencies corresponding to *c*_f_ value of 0.5, 5.0, 10.0. (**d**) Photoacoustic wave amplitude versus frequency at three points located in near field on the cyan dashed ellipse delineated in (**a**–**c**) with polar angles *γ* = 0°, 45°, 90°. (**e**) Photoacoustic wave amplitude versus frequency at three points located at the far field on the ellipse with *ξ*_2_ = 40 also with *γ* = 0°, 45°, 90°. For comparison, the black curve plots the results at the radial distance of 0.145 mm from the equalvolume spherical RBCs with radius of *r* = 2.82 μm. (**f**) The photoacoustic pulses corresponding to the photoacoustic spectral responses shown in (**e**) where the strength for the *γ* = 90° pulse has been reduced by 4 times. To get the results, the bandpass filter with −12 dB bandwidth shown in (**e**) has been applied.

As for the field distributions shown at the left column in Fig. 2(a–c), we have specified their frequencies at *ω*/(2*π*) = 33.4 MHz, 334.0 MHz, 668.0 MHz, which correspond respectively to the *c*_f_ values of 0.50, 5.0, 10.0. The field distributions are calculated in an area of ~30 μm × 30 μm with the RBC sitting at the center where the boundary of the RBC is delineated as the green contour. When frequency goes higher, as shown, the field distribution deviates from the near-isotropic pattern and displays more branches whose shadow edges actually carve out the hyperbolas correlated with spheroidal coordinate *η* (compare to Fig. 1).

This type of frequency-depended behavior is mainly determined by the angle dependent character of angular SWFs. To comprehend it more clearly, in the center column of Fig. 2(a–c), we plotted the polar distributions of the photoacoustic wave amplitude along the *ξ*_1_ = 2.8 ellipse which is delineated as the dashed cyan contour in the field distribution patterns. As can be derived from Eq. (3), the polar distribution is expressed as 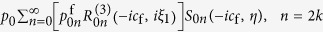. By observing the 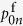 amplitude versus mode number *n* curves plotted in the right column of Fig. 2(a–c) and comparing side by side the polar distributions with the various *S*_0*n*_(−*ic*_f_, *η*) patterns plotted in Fig. S1 (in Supplement), we can see that all of the 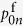 amplitude curves drop very rapidly after a few lowest modes and the *S*_0*n*_(−*ic*_f_, *η*) of these lowest modes determine the polar distributions: for *c*_f_ = 0.5, only the *n* = 0 mode is important; for *c*_f_ = 5.0, the major contribution comes from the *n* = 0, 2 modes; for *c*_f_ = 10.0, the contribution from each of the *n* = 0, 2, 4, 6 modes can be discerned.

The results of Fig. 2(a–c) already imply the fact that the photoacoustic power spectra will vary greatly when measured at different polar angles. In Fig. 2(d), by considering the three points on the *ξ*_1_ = 2.8 ellipse with the corresponding polar angles of *γ* = 0°, 45°, 90°, we plotted their *p*_f_ amplitude versus frequency *ω*/(2*π*) curves (the square of these curves are just their power spectra). Obviously, the values at 33.4 MHz, 334.0 MHz, 668.0 MHz of these curves can also be found in the polar distributions shown in Fig. 2(a–c). As can be seen, these curves overlay with each other at the region below 100 MHz, and then branch out beyond: the red curve for *γ* = 90° is on the top and has a flat shape with a minimum at around 750 MHz, and the green curve for *γ* = 45° and the blue curve for *γ* = 0° lower their magnitude in order but show more and more periods of undulations. We have compared the corresponding power spectra at *γ* = 0°, 90° with those calculated by FEM reported in reference^13^ (refer to the fourth figure therein) and found they are respectively identical after a scaling constant being taken out.

It is interesting to note that these distinguishing structural features shown in Fig. 2(d) are still presented in the far-field spectral amplitude curves, as shown in Fig. 2(e). Here, the *p*_f_ amplitude versus frequency *ω*/(2*π*) curves are plotted for the three points also at *γ* = 0°, 45°, 90° but now on the *ξ*_2_ = 40 ellipse (corresponding to a radial distance ~0.15 mm). For comparison, the result for the equivolume spherical RBC is also plotted, as the black curve.

As a step further from the results of Fig. 2(e), we then calculated the photoacoustic pulses by using the inverse Fourier transform and shown them in Fig. 2(f). For the calculation, we have considered the band-limited frequency response of the ultrasound transducer and have applied a bandpass filtering with −12 dB bandwidth from 200–550 MHz centered at 375 MHz^14^. We found that the results for *γ* = 0°, 90° agree well with the experiment outcomes reported in reference^14^ (the fourth figure there).

### MCF7 cell nuclei surrounded by cell plasma

**Figure 3:**
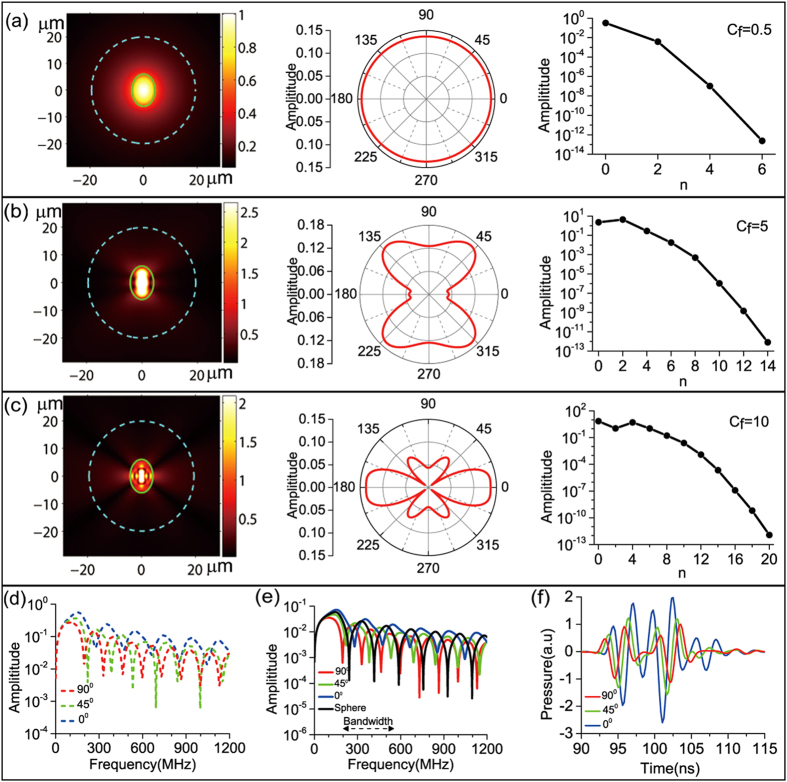
Numerical results of simulating MFC7 cell nuclei suspended in cell plasma. (**a**–**c**) Field distribution, polar distribution, and amplitude of the expansion coefficient 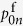 at three different frequencies corresponding to *c*_f_ value of 0.5, 5.0, 10.0. For the field distributions of *c*_f_ = 5.0, 10.0, in order to visualize the parts outside the MFC7 cell nuclei, all of the amplitudes larger than the respective half-maximums of the whole field have been set to those respective half-maximums. (**d**) Photoacoustic wave amplitude versus frequency at three points located in near field on the cyan dashed ellipse delineated in (**a**–**c**) with polar angles *γ* = 0°, 45°, 90°. (**e**) Photoacoustic wave amplitude versus frequency at three points located at the far field on the ellipse with *ξ*_2_ = 35.0 also with *γ* = 0°, 45°, 90°. For comparison, the black curve plots the results at the radial distance of 0.15 mm from the equalvolume cell-nuclei with radius of *r* = 4.76 μm. (**f**) The photoacoustic pulses corresponding to the photoacoustic spectral responses shown in (**e**). To get the results, the bandpass filter with −12 dB bandwidth shown in (**e**) has also been applied.

Cell nuclei are usually close to prolate spheroids in shape thus stand as another type of representative beside RBCs. Here we deal with MCF7 cell nuclei as the example and simulate the situation that they are suspended in cell plasma. We have set the physical parameters of the nucleus as listed also in Table 1 (explained in Method). The cell plasma has been treated as the pure water which has the mass density and acoustic speed under 37 °C respectively as *ρ*_f_ = 1000 kg/m^3^, *v*_f_ = 1527 m/s. We presented various results in Fig. 3 exactly following the way to plot Fig. 2 such that the field distribution, the polar distribution, and the 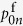 amplitude versus *n* curves are respectively plotted at the left column, the center column, and the right column of Fig. 3(a–c), while the near field and the far field *p*_f_ amplitude versus *ω*/(2*π*) at *γ* = 0°, 45°, 90° are respectively plotted in Fig. 3(d,e), and the corresponding photoacoustic pulses of the far field are plotted in Fig. 3(f).

In order to keep the calculation still at *c*_f_ = 0.5, 5.0, 10.0 in Fig. 3(a–c), the frequencies have been changed to *ω*/(2*π*) = 27.9 MHz, 278.6 MHz, 557.2 MHz. What also changed are the ellipses used for calculating the near field results and the far field results, where now the former has been changed to one with *ξ*_1_ = 4.6 and the latter the other with *ξ*_2_ = 35.0. With the latter setting, the radial distance corresponding to the far field of Fig. 3(e,f) is still ~0.15 mm.

Comparing Fig. 3(a–c) to Fig. 2(a–c), as can be seen, except for *c*_f_ = 0.5 where the field distribution and the polar distribution are still nearly isotropic, the field distributions and the polar distributions for *c*_f_ = 5.0, 10.0 both respectively show significant changes. These changes come from the alteration of the angular SWFs as shown Fig. S2 (in Supplement). We can observe that the modes affording dominate contribution for *c*_f_ = 0.5, 5.0, 10.0 are still respectively *n* = 0, *n* = 0, 2, *n* = 0, 2, 4, 6.

Comparing Fig. 3(d,e) to Fig. 2(d,e), we can see more periodic undulations presenting in the spectral amplitude curves for each of the polar angles. The comparison between Figs 3(f) and 2(f) shows that the strength of the photoacoustic pulses at *γ* = 0°, 45°, 90° are close to each other for the MCF7 cell nuclei, which is significantly different from those of RBCs where the *γ* = 90° photoacoustic pulse strength are about 4 times stronger than the other two.

## Discussion

Before carrying out the detail calculation for the RBCs and the MFC7 cell nuclei, we have first verified the numerical method through studying the two special cases. The verification with a spherical droplet is trivial since it just tests that the radial and angular SWFs will asymptotically reduce to Bessel and Legendre functions respectively. However, the verification with a spheroidal droplet having identical mass density and acoustic speed as those of the surrounding medium is substantial because the spheroid boundary still separates the light absorption region from the surrounding medium thus the SWFs are fully functioning.

The numerical method has been further confirmed by comparing the RBCs power spectra to that calculated previously by FEM. Actually, there are other evidences embedded in Figs 2 and 3. The first is that in the field distributions the pressure amplitudes appears continuous at the spheroid boundary. And the other is that the 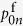 amplitudes decrease very rapidly with mode number *n* which proves the numerical calculation convergence under the truncation mode number setting of Eq. (15).

The results of Figs 2(a–c) and 3(a–c) revealed that the field distribution can be decomposed into different modes, while the results of Figs 2(d,e) and 3(d,e) demonstrated that the undulation structure of power spectra depends on the relative size of the spheroid respect to the wavelength, *i.e.* 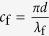. In contrast, the FEM calculation is difficult to unveil these kinds of physical information. Another advantage of the numerical calculation based on SWFs comparing to the FEM calculation is the great flexibility such that the photoacoustic response at any spatial point can be calculated independently, as shown in Figs 2(d–f) and 3(d–f). In the FEM calculation of the photoacoustic response, all of the points inside the whole calculation volume interwind with each other. The above advantages also stand comparing with the *k*-space numerical method^26^ which requires fewer number of spatial grids and time steps than the FEM calculation.

From the results of Figs 2 and 3, we can infer two methods for distinguishing the morphology of a spheroidal biological particle. One method, inferred from the characteristic field distribution at high frequency, is to map the entire photoacoustic field surrounding the particle under the continue wave laser excitation at a high modulation frequency. The other method, inferred from the angular dependent power spectra, is to capture simultaneously the photoacoustic pulse responses at several polar angles under the short pulse laser excitation and then extract the power spectra through the Fourier transform, where the measurement can be performed either in the near field or in the far field. We think the second method can be conveniently incorporated with the flow cytometry to form a new modality for fast spheroidal biological particle sizing. For the case that the rotational axis of each particle is randomized, the above measurement should be taken at more angles distributed in three-dimensions in order to extract first the particle orientation from the symmetry of the measuring results. We note that the spheroidal model calculation provides a good approximation, but the more complex model such as FEM is still necessary to take account the detail particle shape such as the bi-concave shape of RBCs when higher accuracy is required.

As shown in Figs 2(d,e) and 3(d,e), our current calculation is in the range of 0–1200 MHz, corresponding to the maximum of *c*_f_ = 18.0 for the RBCs case and *c*_f_ = 21.5 for MCF7 cell nuclei case. For the latter case, the range can actually be further extended well to *c*_f_ = 36.0. Such limitation ranges of *c*_f_ are due to the numerical capability of the computer package we used^27^,^28^, and can be relaxed by using other advanced program running in multiple-precision algorithm^29^,^30^,^31^. Nevertheless, this frequency range already matches with the cutting-edge high frequency photoacoustic technology^14^,^15^ and is sufficient for the study of biological particles in micrometer scale. We have not considered the sound dispersion in this frequency range since the acoustic speed variation due to the effect is usually only a few percent^19^.

In summary, we have developed a numerical method based on SWFs which can characterize the photoacoustic waves produced by a spheroidal biological particle. This method will be very useful for studying morphology of biological particles which are in spheroidal shape in common. It will be very interesting to rigidly test the numerical calculation by directly comparing to the experiment performed with the artificially created spheroidal droplet. The measurement is not necessarily for a micrometer droplet in the high frequency range since it is the ratio between the droplet size and the wavelength that really matters.

## Methods

### Analytic Theory

Our numerical calculation is rooted on the analytical theory we recently developed for the photoacoustic wave generation from a spheroidal droplet^20^. Here we summarize the key steps.

The analytic theory focuses on solving the following photoacoustic Helmholtz equation where the uniform-heating laser source term has been excluded:


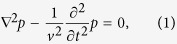


where *p* represents the acoustic pressure produced from the photoacoustic effect, and *v* represents the acoustic speed either inside or for outside the spheroidal droplet.

Equation (1) can be solved in spheroidal coordinates (*ξ*, *η*, *φ*)^21^, and after applying the symmetry restriction conditions, the following reduced expressions in the frequency domain are resulted:


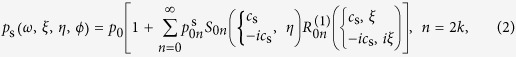



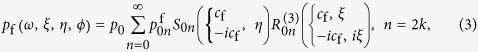


where *p*_s_ and *p*_f_ refer respectively to the photoacoustic waves inside the spheroidal droplet and in its surrounding fluid. The added term *p*_0_ in Eq. (2), coming from the special solution with the laser source term included in Eq. (1), is


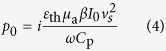


where *I*_0_ and *ω* represents respectively the amplitude and the frequency of the modulated laser intensity, while *μ*_a_ represents the light absorption coefficient, *ε*_th_ the percentage of the absorbed light energy being converted to heat, *C*_p_ the specific heat capacity, *β* the thermal expansion coefficient, and *v*_s_ the sound speed, all for the spheroidal droplet.

In Eqs (2) and (3), *S*_0*n*_ are the angular SWFs while 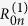 and 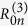 are respectively the first and third radial SWFs, all taking the mode number *m* (related to the azimuthal angle) as zero. The branch symbols for these SWFs are applied to differentiate the variable dependences where the up ones correlate to the prolate spheroidal coordinates system and the bottom ones correlate to the oblate spheroidal coordinates system. The dimensionless variables *c*_s_ and *c*_f_ of these SWFs are defined as


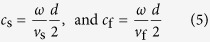


with *d* the interfocal distance of the spheroid droplet and *v*_f_ the acoustic speed of the surrounding fluid.

The expansion coefficients 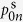 and 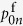 in Eqs (2) and (3) are solved by applying the boundary conditions which require the continuity both in pressure and normal acceleration at the boundary determined by *ξ* = *ξ*_0_ 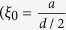 for a prolate spheroid droplet while 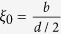 for an oblate spheroidal droplet where *a* and *b* take the conventional notation as the lengths of semi-major and semi-minor axes of a spheroid). The boundary conditions lead to the linear algebra equations as follows


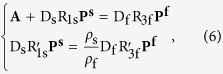


where the column-vectors of **P**^**s**^ and **P**^**f**^ are constituted respectively by 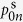 and 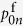 as their elements. Here, 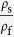 is the ratio between mass densities of the spheroidal droplet and the surrounding fluid, **A** is a constant column-vector with its elements simply as


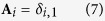


where *δ*_*i*,*j*_ represents the Kronecker delta symbol, all of R_1s_, R_3f_, 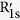, 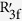 are diagonal square-matrixes formatted respectively as


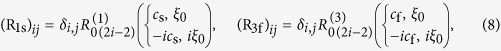



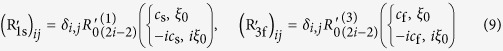


where 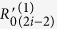 and 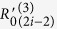 represent the first derivative of 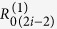 and 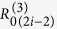 both respective to *ξ*, and both of D_s_ and D_f_ are square-matrixes formatted as


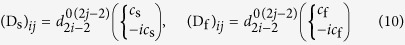


where 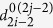 represent the spheroidal coefficients^21^.

Once the frequency domain solution of Eqs (2) and (3) is achieved, its inverse Fourier transform provides the time domain solution.

### Numerical Calculation

As expressed in Eqs (2) and (3), for a prolate or an oblate spheroidal droplet with specified size (i.e. *a* and *b*, thus also *d*), the photoacoustic response in any spatial point depends on not only the values of *S*_0*n*_ and 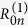 or 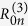 at the specific *η* and *ξ* corresponding to that point, but also the expansion coefficients 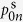 and 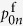 which in turn depends on the value of spheroidal coefficients 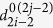 and also the value of 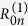, 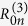, 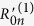, 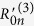 though at this time at *ξ*_0_ corresponding to the boundary [Eqs (6)–(10)]. Therefore, the prerequisite for carrying out the numerical calculation is the computer program which can calculate all of the SWFs (also the spheroidal coefficients).

Up to date, there are already several well-established computer packages for calculating SWFs^27^,^28^,^29^,^32^,^33^,^34^, and some continuous progress in algorithm to improve the accuracy and to expand the range of the *c* parameter is still ongoing^30^,^31^,^35^. In this paper, we choose the computer package running in Matlab^28^ which is translated from the conventional Fortran program^27^. We have not used the more sophisticated computer packages written in Mathematica^29^,^33^ since we only deal with the SWFs of integer modes (actually *m* = 0 and *n* = 2*k*) and the real *c* parameter (since the effect of sound absorption is neglected).

There leaves another issue which is how to numerically solve 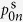 and 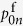, i.e. **P**^**s**^ and **P**^**f**^, from Eq. (6). We combine the two matrix equations in Eq. (6) into a single one which is


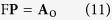


with


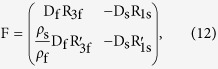



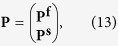



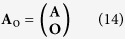


where **O** represents a null column vector with the dimension same as **A**. Now the problem is reduced to calculate **P** from Eq. (11).

Before the numerical calculation can be initiated, however, there is still a requirement to determine the truncation number of the expansion in Eqs (2) and (3). This is equivalent to the request of presetting the dimensions of **P**^**s**^ and **P**^**f**^ thus the dimensions of **P**, F, **A**_o_. . We set the truncation number by referring to the criteria used in reference^36^, as


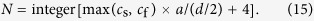


To numerically solve **P** from the dimension-truncated Eq. (11), for the case when the matrix F is ill-conditioned, the advanced “refined iteration method”^36^ has to be excised. However, in our current study, we found that directly calculating the inverse of F by the Gaussian elimination method is sufficient for obtaining the accurate results. We also performed the empirical test on the condition of Eq. (15) and found this setting is sufficient for achieving the convergence.

**Figure 4:**
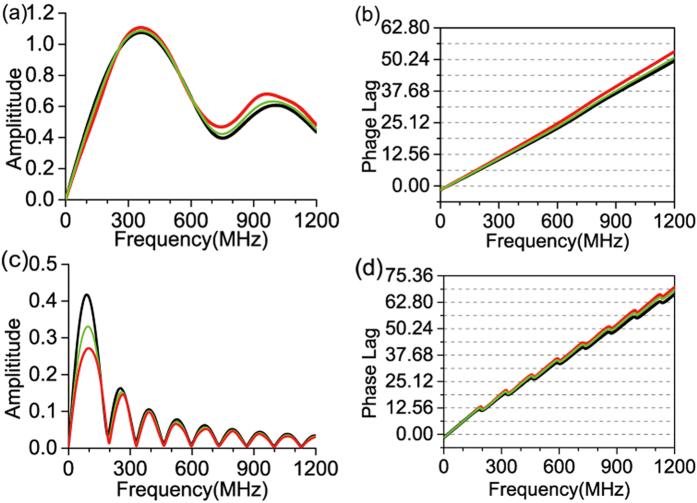
Calculated curves of pressure amplitude versus frequency for RBCs and MFC7 cell nuclei at an outside point on the rotation axis (with *z* = 10.0 μm for RBCs and *z* = 20.0 μm for MFC7 cell nuclei) under various settings of the mass density *ρ*_f_ and acoustic speed *v*_f_ of the surrounding medium. (**a**,**b**) Results for RBCs. The black curve presents the result when *ρ*_f_ and *v*_f_ are the same as those of RBCs (in Table 1). The green curve presents the result when *ρ*_f_ and *v*_f_ are slightly smaller which are *ρ*_f_ = 1077 kg/m^3^, *v*_f_ = 1602 m/s. The red curves presents the result of simulating the RBCs suspended in blood plasma where *ρ*_f_ = 1000 kg/m^3^, *v*_f_ = 1520 m/s. (**c**,**d**) Results for MFC7 cell nuclei. The black curve presents the result when *ρ*_f_ and *v*_f_ are the same as those of MFC7 cell nuclei (in Table 1). The green curve presents the result when *ρ*_f_ and *v*_f_ are slightly smaller which are *ρ*_f_ = 1177 kg/m^3^, *v*_f_ = 1536 m/s. The red curves presents the result of simulating the MFC7 cell nuclei surrounded by cell plasma where *ρ*_f_ = 1000 kg/m^3^, *v*_f_ = 1527 m/s.

There is a final note about the normalization we applied for obtaining the results shown in Figs 2, 3, 4. First of all, since the concrete values of the constants *I*_0_, *ε*_th_, *μ*_a_, *β*, *C*_p_ are not the concern in the current study, we set all of these parameters equal to 1. Then, for each spheroidal droplet, we first calculated the result at the apex point on the major-axe of the spheroid (refer to Fig. 1), and then took its reciprocal as the normalization factor throughout.

### Biological Particle Parameter Settings

We perform the numerical calculation on two representative types of biological particles, namely red blood cells (RBCs) and MCF7 cell nuclei, which can be modeled respectively as oblate spheroidal droplets and prolate spheroidal droplets.

Table 1 lists the size, mass density, and acoustic speed of the RBCs and MCF7 cell nuclei we used in the numerical calculation. As for the RBCs settings, we have referred to references^13^,^14^. As for the MCF7 cell nuclei settings, we have referred to reference^25^ for the size, referred to reference^37^ for the mass density, and referred to reference^38^ for the acoustic speed. Since in reference^38^ only the average acoustic speed across the whole cell is provided, we assumed the acoustic speed of cell plasma as *v*_f_ = 1527 m/s (at 37 °C) and took a simple calculation to extract the acoustic speed of cell nucleus (simply by equaling the time across the whole cell with the summed time sequentially across the cell plasma, cell nuclei, and the cell plasma again).

### Numerical verification

To validate the numerical calculation method, we calculated the spectral response for two special cases. The first is simply when the droplet is a sphere such that the numerical results can be directly compared to those obtained from the standard spherical model^17^. The second is when the droplet is a special spheroid under the condition that its mass density and acoustic speed are respectively identical to those of the surrounding medium, for which the results on the rotational axis can be compared with those obtained from the geometrical calculation method we recently developed^39^.

For the first special case, as shown as the black curves in Figs 2(e) and 3(e), we calculated the pressure amplitude versus frequency of two spherical droplets which have their volume respectively equal to RBCs and MCF7 cell nuclei. We found that these results completely overlay with the outcomes directly calculated from the standard spherical model expression.

For the second special case, we calculated the results of RBCs and MCF7 cell nuclei and plotted them as the black curves in Fig. 4 which including the pressure amplitude versus frequency as well as the pressure phase lag versus frequency. These results are found to be identical with the outcomes of the geometrical calculation method. In the geometrical calculation method, it is the time domain solution of the photoacoustic pulse that can be explicitly expressed, and the frequency domain response is further determined through the Fourier transform.

In Fig. 4, we also plot two other results shown as the green curves and the red curves which come from by gradually breaking the mass density and acoustic speed matching condition to gradually decrease the mass density and the acoustic speed of the surrounding media. As can be seen, the deviation of the red curves from the black curves is larger than the deviation of the green curves from the black curves. These extra comparisons strengthen the verification in a visible way.

## Additional Information

**How to cite this article**: Li, Y. *et al.* Simulating photoacoustic waves produced by individual biological particles with spheroidal wave functions. *Sci. Rep.* **5**, 14801; doi: 10.1038/srep14801 (2015).

## Supplementary Material

Supplementary Information

## Figures and Tables

**Table 1:** Parameter setting for the RBC and the MFC7 cell nucleus.

	*a*	*b*	*d*	*ξ*_0_	*ρ*_s_ (37 °C)	*v*_s_ (37 °C)
RBC	3.91 μm	1.47 μm	7.24 μm	0.40	1110 kg/m^3^	1650 m/s
MFC7 cell nucleus	6.03 μm	4.22 μm	8.61 μm	1.40	1430 kg/m^3^	1582 m/s
