# Supplementary material for: Simulating photoacoustic waves produced by individual biological particles with spheroidal wave functions
Source: Sci Rep. 2015 Oct 7;5:14801. doi: 10.1038/srep14801 (PMC4595827; doi:10.1038/srep14801)
Supplement: Supplementary Information [file srep14801-s1.pdf]

Supplementary Figures for

Simulating photoacoustic waves produced by individual biological particles with spheroidal wave functions

Yong Li[1], Hui Fang[2,*], Changjun Min[2], and Xiaocong Yuan[2]

[1]Institute of Modern Optics, Key Laboratory of Optical Information Science and Technology, Ministry of Education of China, College of Electronic Information and Optical Engineering, Nankai University, Tianjin 300071, China

[2]Institute of Micro and Nano Optics, Key Laboratory of Optoelectronic Devices and Systems of Ministry of Education and Guangdong Province, College of Optoelectronic Engineering, Shenzhen University, Shenzhen 518060, China

[*]fhui79@szu.edu.cn

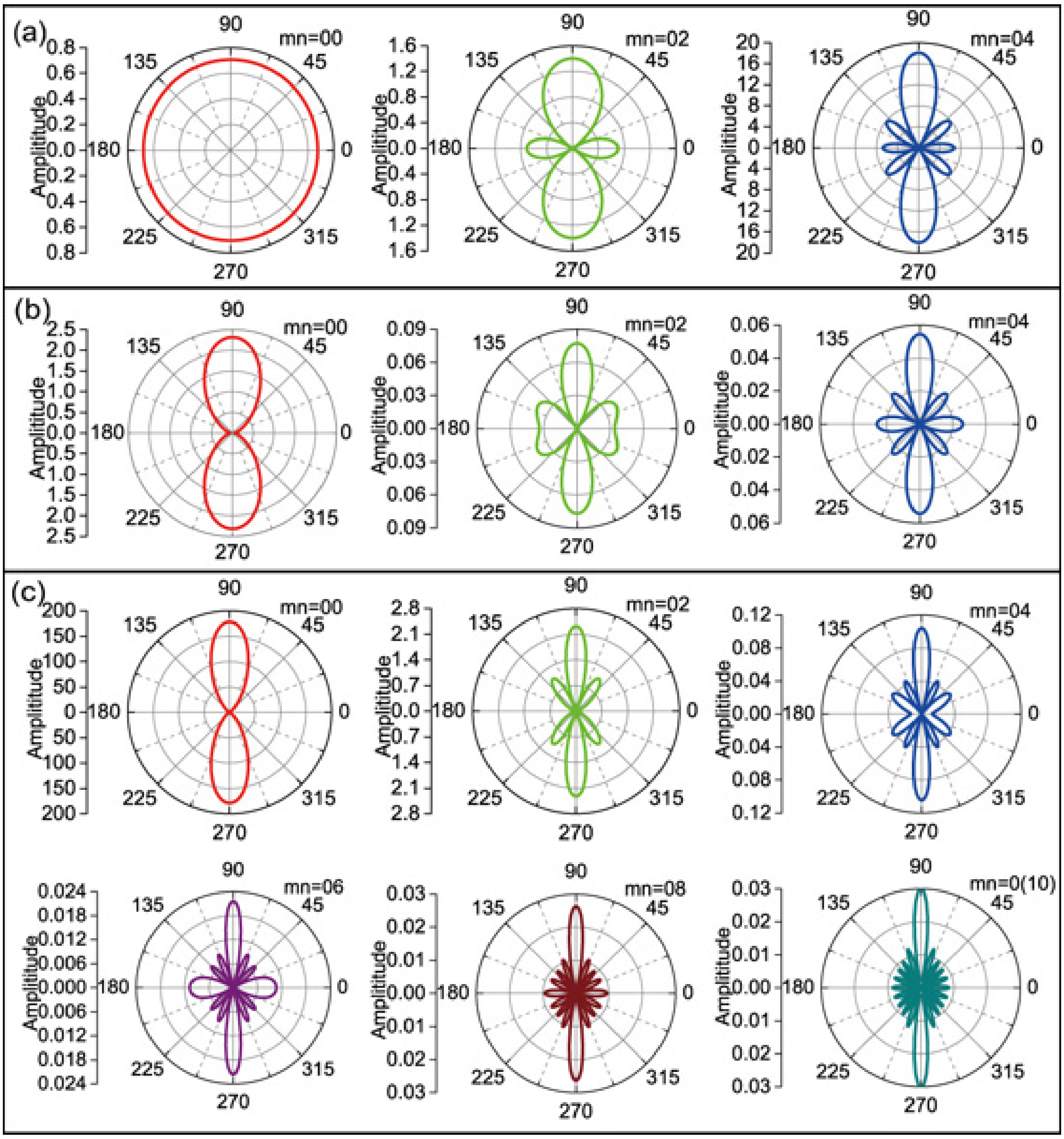

Fig. S1 The polar distribution patterns for a few lowest modes angular SWFs of oblate spheroids at three typical frequencies corresponding to $c_f = 0.5, 5.0, 10.0$. These results can be compared to the standard values listed in [1, 2].

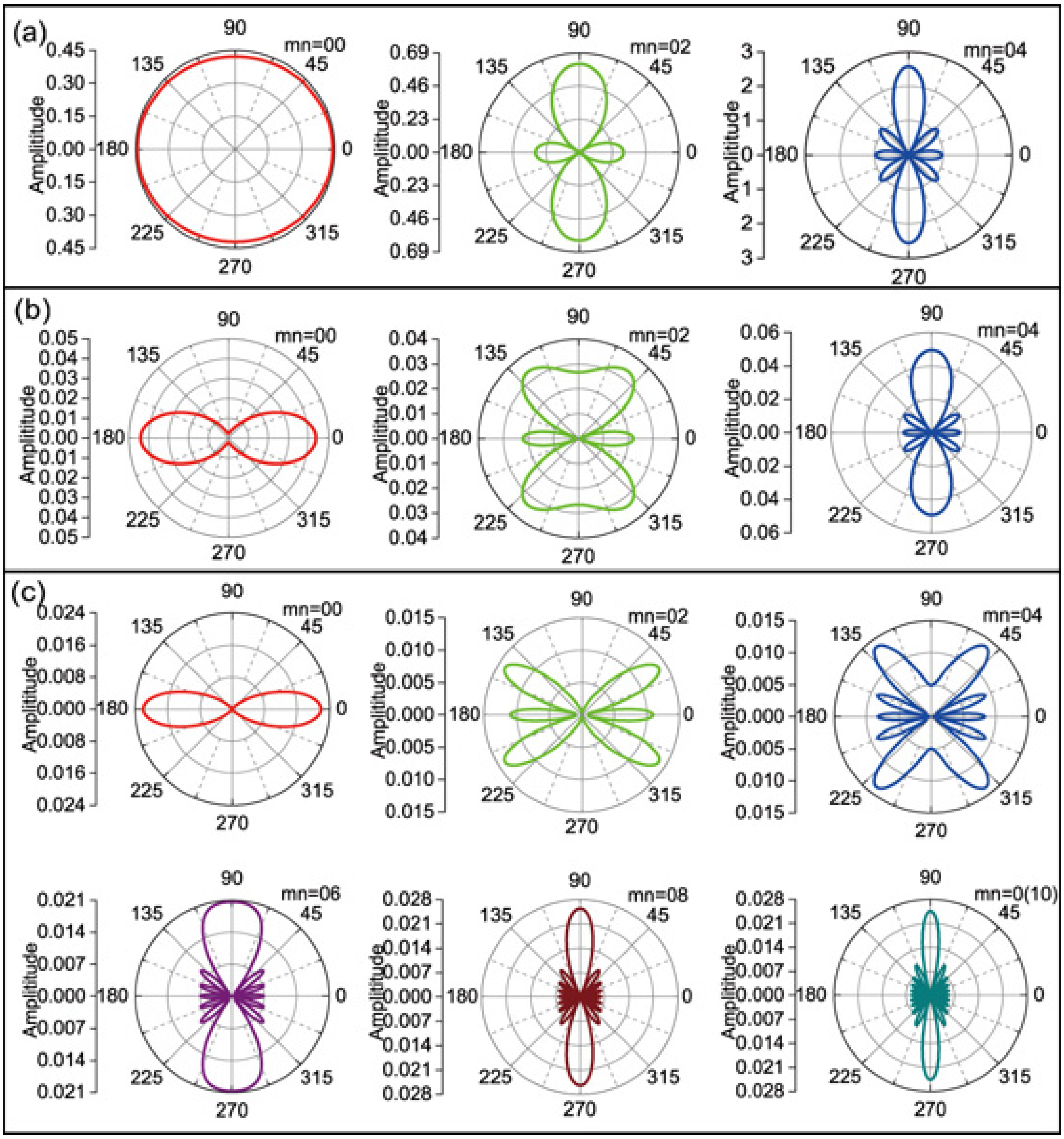

Fig. S2 The polar distribution patterns for a few lowest modes angular SWFs of prolate spheroids at three typical frequencies corresponding to $c_f = 0.5, 5.0, 10.0$. These results can be compared to the standard values listed in [1, 2].

References

[1] C. Flammer. *Spheroidal Wave Functions* (Stanford University Press, Stanford, CA, 1957).

[2] S. Zhang, and J. Jin. *Computation of Special Functions* (Wiley, New York, 1997).
